# The Effect of Transcranial Random Noise Stimulation on Cognitive Training Outcome in Healthy Aging

**DOI:** 10.3389/fneur.2021.625359

**Published:** 2021-03-09

**Authors:** Michela Brambilla, Lars Dinkelbach, Annelien Bigler, Joseph Williams, Nahid Zokaei, Roi Cohen Kadosh, Anna-Katharine Brem

**Affiliations:** ^1^Department of Experimental Psychology, University of Oxford, Oxford, United Kingdom; ^2^Biomedical and Clinical Sciences Department, Center for Research and Treatment on Cognitive Dysfunctions, “Luigi Sacco” Hospital, University of Milan, Milan, Italy; ^3^Department of Neurology, Institute of Clinical Neuroscience and Medical Psychology, Heinrich Heine University, Duesseldorf, Germany; ^4^Department of Experimental Psychology, Ghent University, Ghent, Belgium; ^5^Medical Sciences Division, University of Oxford, Oxford, United Kingdom; ^6^Department of Neurology, Berenson-Allen Center for Noninvasive Brain Stimulation and Division for Cognitive Neurology, Beth Israel Deaconess Medical Center and Harvard Medical School, Boston, MA, United States

**Keywords:** healthy aging, transcranial random noise stimulation, cognitive training, cognitive enhancement, executive functions

## Abstract

**Background and Objective:** Aging is associated with a decline in attentional and executive abilities, which are linked to physiological, structural, and functional brain changes. A variety of novel non-invasive brain stimulation methods have been probed in terms of their neuroenhancement efficacy in the last decade; one that holds significant promise is transcranial random noise stimulation (tRNS) that delivers an alternate current at random amplitude and frequency. The aim of this study was to investigate whether repeated sessions of tRNS applied as an add-on to cognitive training (CT) may induce long-term near and far transfer cognitive improvements.

**Methods:** In this sham-controlled, randomized, double-blinded study forty-two older adults (age range 60–86 years) were randomly assigned to one of three intervention groups that received 20 min of 0.705 mA tRNS (*N* = 14), 1 mA tRNS (*N* = 14), or sham tRNS (*N* = 19) combined with 30 min of CT of executive functions (cognitive flexibility, inhibitory control, working memory). tRNS was applied bilaterally over the dorsolateral prefrontal cortices for five sessions. The primary outcome (non-verbal logical reasoning) and other cognitive functions (attention, memory, executive functions) were assessed before and after the intervention and at a 1-month follow-up.

**Results:** Non-verbal logical reasoning, inhibitory control and reaction time improved significantly over time, but stimulation did not differentially affect this improvement. These changes occurred during CT, while no further improvement was observed during follow-up. Performance change in logical reasoning was significantly correlated with age in the group receiving 1 mA tRNS, indicating that older participants profited more from tRNS than younger participants. Performance change in non-verbal working memory was significantly correlated with age in the group receiving sham tRNS, indicating that in contrast to active tRNS, older participants in the sham group declined more than younger participants.

**Interpretation:** CT induced cognitive improvements in all treatment groups, but tRNS did not modulate most of these cognitive improvements. However, the effect of tRNS depended on age in some cognitive functions. We discuss possible explanations leading to this result that can help to improve the design of future neuroenhancement studies in older populations.

## Introduction

“Cognitive health” is consistently cited as an essential factor contributing to functional ability and quality of late life (1, 2). Cognitive decline along with physiological, structural, and functional brain changes represents a crucial component of healthy aging (3, 4). The frontal lobes are particularly vulnerable to age-related deterioration, potentially explaining the most important changes in cognitive performance associated with normal aging; these changes primarily affect cognitive activities that require rapid information processing, such as working memory and other executive functions (5). Age-related decline is thought to be actively counteracted by the brain with compensatory processes, as can be seen, for example, in positive associations between the activation of bilateral frontal regions and cognitive performance (1, 6, 7). The frontal lobes have therefore been singled out as a potential target for early interventions to counteract age-related changes and maintain cognitive function (8–10).

Neuroenhancement describes the use of neuroscience-based techniques to enhance cognitive function, acting directly on the human cortex to alter its properties and increase performance for a specific cognitive task or a set of tasks (11). A wide variety of neuroenhancement methods have been developed in the last decade, one of them being transcranial electrical stimulation (tES). tES approaches are non-invasive neuromodulatory techniques that use the application of electrical current over the scalp, to facilitate or inhibit spontaneous neuronal activity resulting in altered brain functions. tES with its easy and safe application has the potential to serve as a cognitive enhancer in healthy older populations, as well as a therapeutic intervention to compensate for deficits in individuals with neurological and psychiatric conditions (12–14). Recent meta-analyses and systematic reviews provide robust support for enhancing both cognitive and motor performance in healthy aging with direct current stimulation (tDCS) in single and multiple sessions (15–17). A more recently developed type of tES is transcranial random noise stimulation (tRNS) that delivers an alternate current at random amplitude and frequency (18). Moliadze et al. showed that tRNS could elicit a more pronounced amplitude elevation of motor evoked potentials than tDCS (19). However, these results have not been universally supported, with others finding no clear difference between the effects of tDCS and tRNS on motor cortex excitability (20). tRNS might lead to more widespread effects via stochastic resonance (21) and the aging brain might react differently to tRNS due to the activation of broader networks (22). Age appears to be associated with more beneficial outcomes in neurostimulation in some studies (23, 24). One possible explanation is that lower performing individuals are thought to profit more from cognitive interventions (25) and neurostimulation (26) and show more consistent stimulation gains (27). However, other studies demonstrated that differences in education level may account for the differing effects of neurostimulation between age groups (28).

Since the pioneering study on CT, the CT ACTIVE trial in older adults by Ball et al. (29), several studies assessing intensive cognitive rehabilitation approaches targeting attention, information processing speed, memory, and executive function reported positive results in older adults. Most reported a behavioral improvement with “transfer” to other tasks and functional ability at the end of a training period; a few studies focusing on the neurophysiological changes underlying this improvement showed increased frontal brain activity after training (30–32). However, recent meta-analyses indicate that there is a lack of clear-cut findings regarding the long-term efficacy and generalizability of such programs (33–37). Recently, researchers have started to combine CT with neurostimulation. The rationale for combining approaches is derived from the assumption that combinatory approaches, such as neurostimulation and CT, are more effective in achieving long-lasting effects than each approach on its own. The application of tES alone does not lead to neuronal depolarization, but is thought to modulate the resting membrane potential either toward or away from the depolarization threshold. Within this framework, intrinsic fluctuations, e.g., resulting from a concomitant task, are more likely to lead to neuronal firing when the resting membrane potential is shifted toward the threshold via application of an external modulator (i.e., tES) (38). Through this mechanism neurostimulation is thought to enhance ongoing brain processes and therefore improve training-related effects (39).

To date, only few studies investigated the combined effect of repeated tDCS sessions combined with CT on healthy aging participants (40–42). In the study by Meinzer et al. (40), subjects acquired a novel vocabulary over 5 consecutive days and received either tDCS over the left posterior temporo-parietal junction or sham tDCS. TDCS yielded steeper learning curves and enhanced learning success at the end of the training and at 1-week follow-up. Park et al. (41) assessed the effects of ten sessions of tDCS over the bilateral prefrontal cortices during CT on attention and executive functions in healthy older adults. Both verbal working memory and digit span forward improved significantly after CT combined with active tDCS vs. sham tDCS. In the study of Jones et al. (42) participants received ten sessions of sham or active (anodal, 1.5 mA) tDCS to the right prefrontal, parietal, or prefrontal/parietal (alternating) cortices and afterwards performed a short verbal and visual working memory training. All groups benefited from working memory training, however, at the 1-month follow-up only participants from the active tDCS groups maintained significant improvement for both trained and transfer tasks. The majority of studies report improvements on near transfer effects (i.e., improvements on tasks that are similar to the trained tasks). To our knowledge, to date only one study reported far transfer effects in older adults. Stephens and Berryhill (43) found far transfer effects on ecologically valid tasks, such as weekly calendar planning and driving knowledge, 1 month after a 5-day intervention involving working memory training and tDCS. Conversely, some studies reported no improvement after tDCS combined with CT (44, 45). Recent studies have attributed the effect of tRNS on cognitive training (CT) in young adults to modulation of neurophysiological mechanisms that are associated with sustained attention (46). Indeed, in children with attention deficit/hyperactivity disorder, tRNS with CT over the dorsolateral prefrontal cortices has yielded promising clinical improvement compared to tDCS with CT (47). Moreover, a recent study in juvenile mice revealed that chronic tRNS over the prefrontal cortex, with similar parameters to what has been used in atypically developing children (48), reduced GABAergic activity (49). Single sessions of tRNS showed promise in modulating cognitive functions in aging (22, 28, 50). However, to date, the potential of tRNS as an effective cognitive enhancer in older adults remains to be shown. For example, in a recent study by Fertonani et al. (22), the authors investigated the neurophysiological mechanism underlying tRNS effects applied over the visual cortex in healthy elderly. They found that tRNS modulated visuo-perceptual learning in young, but not older healthy adults. Moreover, tRNS appears to have differential effects on young and older adults. Cappelletti et al. (50) compared tRNS over the motor and parietal cortex with sham stimulation during CT. While both age groups showed similar improvements in numerosity discrimination, these improvements were driven by different mechanisms and led to different transfer effects in the two age groups.

The aim of the present study was to investigate whether repeated sessions of bilateral tRNS applied at one of two different intensities (1 mA or 0.705 mA) over the dorsolateral prefrontal cortices applied as an add-on to executive function training induces immediate and long-term cognitive improvements compared to sham stimulation combined with executive function training. We hypothesized that active stimulation vs. sham would result in increased frontal connectivity during executive function training in older adults resulting in near and far transfer cognitive improvements.

## Materials and Methods

### Participants

Fifty healthy right-handed participants aged 60 or older were enrolled in the study of which forty-seven underwent all study measures (see Table 1 for demographic characteristics). Two subjects dropped out after baseline measures due to personal commitments. One person (receiving 1 mA tRNS) dropped out after the second intervention visit due to headache. Participants were recruited via the website *www.joindementiaresearch.co.uk*. Exclusion criteria were contraindication to brain stimulation (51), current history of drug or alcohol abuse or dependence, any past or present psychiatric or neurological disorder, and subjective cognitive complaint. Participants performed the Montreal Cognitive Assessment (MoCA) (52, 53) and Beck Depression Inventory-II (BDI-II) (54) at the pre-test session to screen for cognitive impairment and depression; participants with a score lower than 24/30 in the MoCA (55) or with a score higher than 18/63 in the BDI-II were excluded from the study. All subjects received detailed information about the study protocol and voluntarily gave their written informed consent prior to study commencement. Participants were naive with respect to the experimental hypothesis and remained unaware of what type of stimulation they received. The study was approved by the ethics committee of the University of Oxford and performed in accordance with the Declaration of Helsinki. Participants were instructed to sleep at least 6 h each night, abstain from alcohol for the study duration, and refrain from caffeine for 1 h before study visits.

**Table 1:** Demographic characteristics of individuals grouped according to experimental condition.

**Demographic characteristics**	**Sham**	**Active 1 mA**	**Active 0.705 mA**	***p*-Value**	**Partial eta squared**
Number of participants	19	14	14		
Gender (male/female)	11/8	8/6	7/7	0.919	
Age (years)	69.4 (6.3)	69.4 (5.8)	69.2 (8.7)	0.996	<0.001
Education (years)	15.6 (2.6)	16.6 (3.3)	17.1 (3.3)	0.347	0.047
MoCA	27.1 (1.9)	27.5 (1.5)	28.4 (1.3)	0.067	0.116

*Raw scores and standard deviations are reported. MoCA, Montreal Cognitive Assessment*.

### Study Design

**Figure 1:**
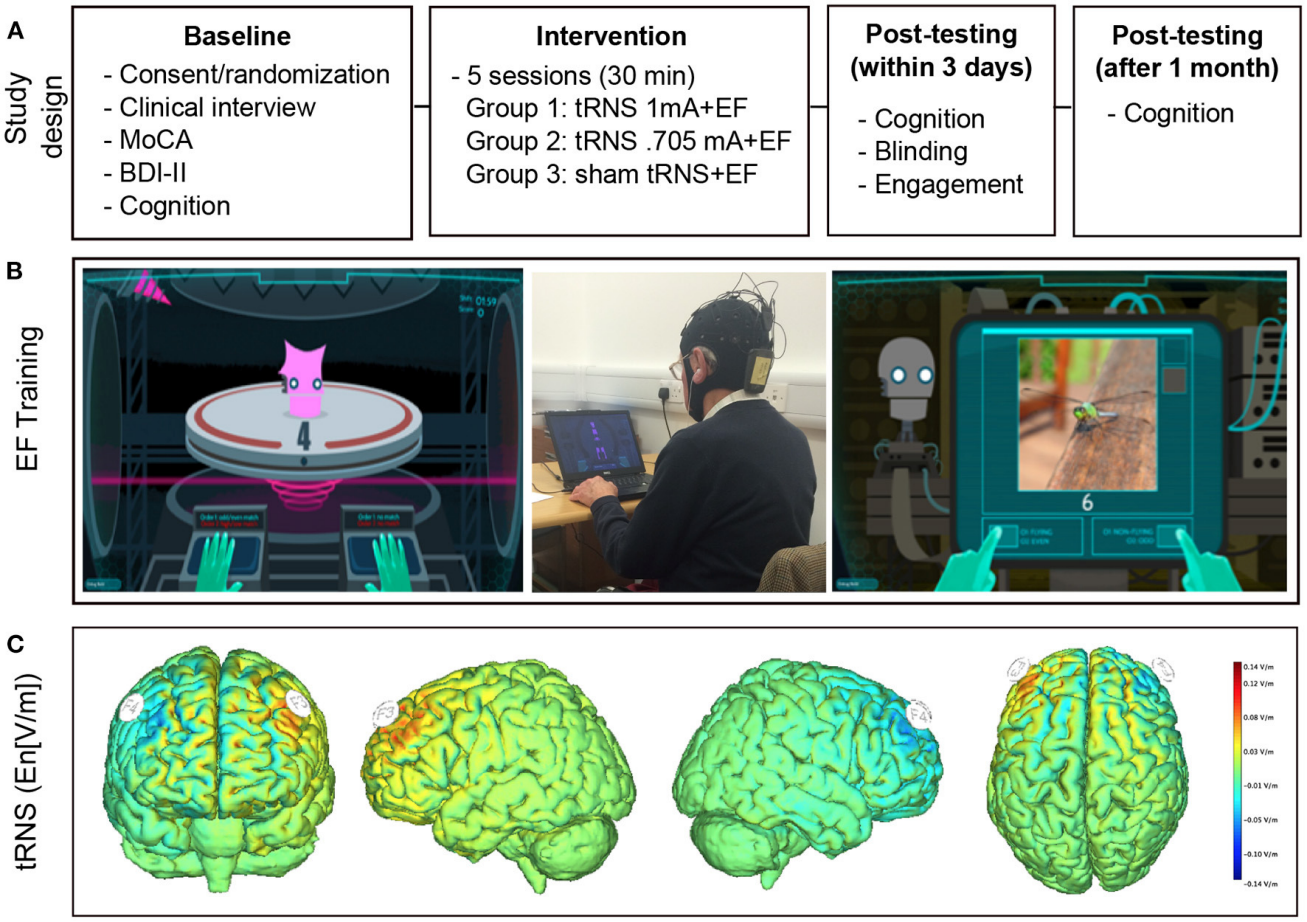
Experimental design and intervention. **(A)** Experimental Design. After baseline testing participants were randomized to receive executive function training combined with real tRNS (1 or 0.705 mA) or sham tRNS. Cognitive tests were repeated immediately after and 1 month after the intervention. **(B)** Cognitive training. Examples of single (left panel) and complex tasks (right panel). The middle panel depicts the intervention setup. **(C)** Modeling of the current flow. Stimulation was delivered over the dorsolateral prefrontal cortex and stimulation onset coincided with training onset. Electric field calculations (component of the electric field orthogonal to the cortical surface En [V/m]) were performed using a realistic head model (56). Positive values indicate that the field is directed into the cortical surface. Note that the protocol only depicts one instance of stimulation given that polarity changes continually during tRNS.

The study design is depicted in Figure 1. In this randomized, sham-controlled, double blind trial, CT was combined with tRNS over five sessions. Sessions took place on consecutive workdays in a block of three (Wednesday, Thursday, Friday) and 2 days (Monday, Tuesday) and were therefore spread across 2 weeks. Participants were randomly assigned to one of three intervention groups: (1) 0.705 mA tRNS plus CT, *N* = 14; (2) 1 mA tRNS plus CT, *N* = 14; and (3) sham tRNS plus CT, *N* = 19. Notably, the original study design included two stimulation intervention groups (1 mA tRNS vs. sham tRNS combined with CT). However, due to a technical error five additional subjects received sham stimulation and a number of subjects were stimulated at a 30% lower intensity level. We therefore decided to adapt the original study design to include a third group receiving this lower stimulation intensity (0.705 mA). The research staff administering the intervention remained blinded. All participants underwent a neuropsychological and experimental assessment at baseline and again within 2 days after the intervention. We decided to test participants' cognitive performance not immediately after the last training session in order to avoid possible cognitive benefits due to immediate stimulation after-effects, or cognitive fatigue immediately after CT. One month later, participants were tested again in order to assess long-term effects of the intervention (Figure 1).

### Cognitive Training

A newly-developed CT program (Flexible, Adaptive, Synergistic Training, FAST, Simcoach Games, Pittsburgh, PA, USA) was used to train participants in a series of gamified isolated and integrated executive function tasks [for a detailed description see (57)]. CT (30 min per session) was presented on a laptop while subjects listened to custom-designed music and game-specific sounds via earphones. Participants were trained in a quiet laboratory environment. The training consisted of a series (15 blocks, each 2 min) of tasks, drawing on working memory (n-back tasks), inhibition, and cognitive flexibility. Each training block was preceded by instructions and followed by a performance feedback. The training was adaptive, i.e., whenever participants passed 80% of a training block, difficulty levels were increased. This way an individually adapted and constant level of challenge was ensured. When 50–80% corrects were reached, the difficulty level was maintained, while difficulty decreased when subjects reached <50% correct answers. The overall training started with exercises of single sub-functions (working memory or inhibition or cognitive flexibility) and advanced to combinations of two or all three sub-functions. Difficulty was furthermore increased by reducing the time available to respond, or by increasing the number of items, while changing training parameters in the opposite direction decreased difficulty.

### Transcranial Random Noise Stimulation

Active and sham tRNS was delivered via gel-filled pi-electrodes (3.14 cm^2^) that were inserted into a neoprene cap (Starstim®, Neuroelectrics, Barcelona, Spain) with predefined localization of the anode (F3) and the cathode (F4). StimWeaver (Neuroelectrics, Barcelona, Spain) was used to optimize the stimulation montage according to the specified targets. Electric field calculations (component of the electric field orthogonal to the cortical surface En [V/m]) were performed using a realistic head model (56). Positive values indicate that the field is directed into the cortical surface. Note that the tRNS protocol only depicts one instance of stimulation given that polarity changes continually during stimulation (Figure 1). According to the 10–20 international EEG system these locations correspond to the left and right dorsolateral prefrontal cortex. During each session, 20 min of high-frequency tRNS (100–640 Hz) at 1 or 0.705 mA or sham stimulation was delivered. During the latter, current was ramped up to a stimulation intensity of 1 mA and then down again over a period of 30 s. Stimulation onset coincided with training onset. Neither the subjects nor the study staff administering the training and testing of cognitive functions were aware about the nature of the stimulation.

### Neuropsychological Assessment

The neuropsychological test battery included a range of standardized and experimental tasks that were completed in a fixed order, with an average duration of 60 min. All tasks are described below in detail. Trained staff, which was blinded to patient intervention allocations, administered the neuropsychological tests. The same assessor administered all assessments (baseline, post-test, and follow-up) for each single subject in order to minimize bias. Far transfer effects were assessed with a non-verbal logical reasoning task [Sandia (58), primary outcome] as well as attention and memory tasks. Near transfer effects were assessed with executive tasks that were dissimilar from the training tasks.

#### Inhibitory Control (False Alarms)

To assess changes in inhibitory control we used a computerized Go/Nogo task programmed in Matlab that was previously used (59). Participants were asked to respond to a centrally located target letter that appeared on a screen. Upon the presentation of the letters X (Go) or O (Nogo) they had to press the respective response keys as quickly as possible. The letters were white on a black background and the stimuli size was 0.7 by 0.7 cm. Each stimulus was displayed for 100 ms followed by a random intertrial interval between 1,000 and 2,000 ms. The stimuli were presented randomly in three blocks of 150 trials and consisted of 70% Go trials and 30% NoGo trials. Subjects were instructed to respond as fast as possible with the index finger of the dominant hand.

#### Working Memory

We used a computerized task (Matlab) to examine spatial working memory. The task measured precision, which provides a more sensitive measure of working memory capacity than traditional span tasks (60). The stimuli were randomly oriented colored bars located centrally on a gray background and always differed at least 10° in orientation from the preceding bar. In this 4-item (high-load) working memory task (Supplementary Figure 1) four bars of random color and orientation were presented. Before each block, participants were instructed to remember the orientation of all bars. After the four stimulus bars were presented, a probe bar of any of the previously presented colors was presented after a 500 ms delay. Participants were asked to rotate the probe bar to the orientation of the same-colored bar from memory. Before starting the main task, participants practiced (30 trials each). The main task included two 30-trial blocks, participants therefore completed 60 trials. For each item, recall performance was defined as the difference between the target and submitted angles (60). Precision was calculated as the circular standard deviation of the error response, with reduced variability showing greater overall precision. Precision is therefore given in 1/radian.

#### Attention (Reaction Time)

We assessed reaction time with a version of the attention network test (ANT). The details of this task are described elsewhere (61). Stimuli were presented via E-prime software on a 17″ monitor, alerting tones were presented through headphones.

#### Verbal Fluency (Semantic and Phonemic)

In this task, the subject was asked to produce orally as many words as possible within 2 min beginning with a specific letter (S, F, or A) or from a specific category (fruit, animals, or clothing). Conditions were randomized between visits.

#### Short-Term Memory and Working Memory

Digit span tasks (span length forward and backward) from the Wechsler Adult Intelligence Scale-Revised (62) as well as blocktapping forward and backward were used to assess verbal and visuospatial short-term memory and working memory.

#### Non-verbal Logical Reasoning

Sandia matrices overcome the issue of a limited number of stimuli by providing the option to choose from a pool of ~3,000 matrices, obtained through the combination of different stimulus features like shape, color and orientation (58). Experimental matrices were chosen from four different classes based on the type and number of analogical operations required for a correct solution (1-, 2-, 3- relations and logic matrices) and matched in terms of their difficulty for a set of three parallel test versions that were randomized across time-points. Participants were given four practice trials before starting that actual task that required them to solve as many matrices as possible within 15 min. This task measuring far transfer effects was defined as the primary outcome.

#### Questionnaires

An adapted version of the questionnaire developed by Jennett et al. (63) was administered after the last session of training to register as how pleasant, interesting, rewarding and meaningful the training was perceived. Its thirty-five items are scored on a scale between 0 and 7 (**Table 3**). Potential side effects, such as perception of itching, burning, etc., were assessed with a questionnaire. Blinding efficacy was determined by asking subjects at the end of the last training session to guess if they had received active or sham stimulation (i.e., “In your opinion, did you receive a real stimulation or a placebo stimulation?”).

### Power Considerations and Data Analysis

Power calculations were performed with G^*^Power 3.1 (64). Assuming an effect size (Cohens'd) of f(V) = 0.6, suggesting a medium effect, a sample size of 42 was estimated to have a 85% power using repeated-measures ANOVA with a 0.05 two-sided significance level. Given that another study with a similar design using tDCS (43) showed a similar effect size, we believe that our estimate was justified. To identify relevant differences between groups in demographic characteristics, univariate ANOVAs were calculated for numerical data (age, MoCA, education). For gender and blinding efficacy, Chi-Square tests were calculated. To identify changes in neuropsychological measures due to neurostimulation, repeated measures ANOVAs for each outcome parameter with time (baseline, post-test, follow-up) as a within-subject and group (sham, 1, 0.705 mA) as a between-subjects variable were calculated and checked for interaction-effects. Violations of the assumption of sphericity where examined with the Mauchly's test and adjusted by the Greenhouse-Geisser correction if necessary. Analyses were conducted using IBM SPSS Statistics 26 (International Business Machines Corporation, Armonk, USA) and JASP.

## Results

None of the recruited participants were excluded in the screening phase. At baseline, no significant differences of age, gender, education, or cognitive abilities as measured with the MoCA were observed (see Table 1).

### Neuropsychological Assessment

The mean and standard deviation of all neuropsychological assessments for all three timepoints (baseline, post-test, follow-up) separated by groups (sham, 1, 0.705 mA) are being presented in Table 2. None of the interaction effects (time^*^group) reached the level of significance, no differential effect of neurostimulation on measures of the neuropsychological assessment was observed in the domains of *Memory* (verbal short-term memory [*F*_(4,86)_ = 0.184, *p* = 0.946, $\eta_{\text{p}}^{2}$ = 0.008]; non-verbal short-term memory [*F*_(4,86)_ = 0.980, *p* = 0.423, $\eta_{\text{p}}^{2}$ = 0.044], *Executive Functions* (phonemic fluency [*F*_(4,88)_ = 0.428, *p* = 0.788, $\eta_{\text{p}}^{2}$ = 0.019]; semantic fluency [*F*_(4,84)_ = 1.245, *p* = 0.298, $\eta_{\text{p}}^{2}$ = 0.056]; verbal working memory [*F*_(4,86)_ = 0.575, *p* = 0.682, $\eta_{\text{p}}^{2}$ = 0.026]; non-verbal working memory [*F*_(4,86)_ = 0.397, *p* = 0.810, $\eta_{\text{p}}^{2}$ = 0.018]; inhibitory control [*F*_(3.09,64.95)_ = 1.075, *p* = 0.367, $\eta_{\text{p}}^{2}$ = 0.049, adjusted by Greenhouse-Geisser]), the primary outcome *Logical Reasoning* (Sandia [*F*_(4,76)_ = 0.457, *p* = 0.767, $\eta_{\text{p}}^{2}$ = 0.023]), and *Attention* (reaction time [*F*_(3.24,66.35)_ = 1.883, *p* = 0.137, $\eta_{\text{p}}^{2}$ = 0.084], adjusted by Greenhouse-Geisser).

**Table 2:** Neuropsychological outcomes at baseline, post-testing, and follow-up.

**Variable**	**Stimulation**	**Baseline**			**Post-test**			**Follow-up**			***df***	***F***	***p***
		***N***	**Mean**	***SD***	***N***	**Mean**	***SD***	***N***	**Mean**	***SD***			
**COGNITIVE SCREENING**													
MoCA	0	19	27.1	1.9									
	0.705	14	28.4	1.3									
	1	14	27.5	1.5									
**MEMORY**													
Verbal STM	0	18	9.94	2.01	19	10.21	1.93	19	10.74	2.35	2.34	2.109	0.137
	0.705	14	11.50	2.21	14	11.57	2.59	14	12.00	2.54	2.26	1.143	0.334
	1	14	11.50	1.74	14	11.64	2.73	14	11.79	2.46	2.26	0.105	0.901
Non-verbal STM	0	18	8.67	1.97	19	8.11	2.02	19	7.63	2.03	2.34	1.755	0.188
	0.705	14	8.57	1.70	14	8.64	1.50	14	8.86	2.14	2.26	0.17	0.845
	1	14	8.14	1.70	14	8.50	1.95	14	7.71	1.49	2.26	0.867	0.432
**EXECUTIVE FUNCTIONS**													
Phonemic fluency	0	19	23.68	6.19	19	22.11	7.19	19	24.05	5.52	2.36	0.919	0.408
	0.705	14	25.43	9.25	14	24.86	6.90	14	25.43	9.01	2.26	0.109	0.898
	1	14	24.50	4.67	14	24.79	6.69	14	26.71	5.28	2.26	1.568	0.227
Semantic fluency	0	18	26.33	7.64	19	26.11	9.09	19	26.74	7.72	2.34	0.115	0.891
	0.705	13	31.54	11.13	14	25.57	8.29	14	25.36	6.40	2.24	2.847	0.078
	1	14	29.29	9.02	14	30.57	6.52	14	28.21	6.58	2.26	0.33	0.722
Verbal WM	0	18	7.44	2.06	19	7.42	2.14	19	7.42	2.24	2.34	0.06	0.942
	0.705	14	8.36	2.13	14	8.43	2.14	14	8.71	3.00	2.26	0.194	0.825
	1	14	7.43	2.24	14	7.79	2.46	14	8.57	2.41	2.26	1.767	0.191
Non-verbal WM	0	18	7.72	1.81	19	7.00	2.21	19	6.79	1.99	2.34	1.289	0.289
	0.705	14	8.00	1.24	14	7.64	1.95	14	7.79	1.63	2.26	0.414	0.665
	1	14	7.43	1.45	14	7.14	1.96	14	7.50	2.44	2.26	0.151	0.861
Visuospatial WM	0	15	0.980	0.137	16	1.025	0.136	16	1.008	0.199	2.28	0.686	0.512
	0.705	11	1.005	0.111	11	1.100	0.254	12	0.971	0.077	2.20	2.261	0.13
	1	13	0.901	0.054	13	0.927	0.098	13	0.968	0.066	2.22	3.223	0.059
Inhibitory control	0	19	0.098	0.066	19	0.072	0.052	19	0.064	0.049	2.36	3.449	0.043
	0.705	13	0.094	0.097	14	0.057	0.060	13	0.055	0.060	2.22	4.462	**0.024**
	1	14	0.084	0.084	14	0.070	0.063	14	0.085	0.066	2.26	0.644	0.533
**ATTENTION**													
Reaction time	0	18	644	74	19	598	92	19	594	79	2.34	7.187	**0.002**
	0.705	13	608	81	14	586	72	13	592	62	2.22	0.63	0.542
	1	14	598	46	14	561	53	14	550	53	2.26	9.301	**0.001**
**LOGICAL REASONING**													
Sandia	0	15	8.87	5.15	18	11.78	5.32	18	11.78	5.84	2.28	8.412	**0.001**
	0.705	12	9.33	4.16	13	10.92	4.11	14	11.86	5.74	2.22	2.811	0.082
	1	14	10.00	3.57	14	11.86	3.37	14	13.36	4.85	2.26	3.801	**0.036**

*Raw scores (mean) and standard deviations (SD) are reported. MoCA, Montreal Cognitive Assessment; STM, short-term memory; WM, working memory. Bold font indicates significant p-values (p < 0.05)*.

**Figure 2:**
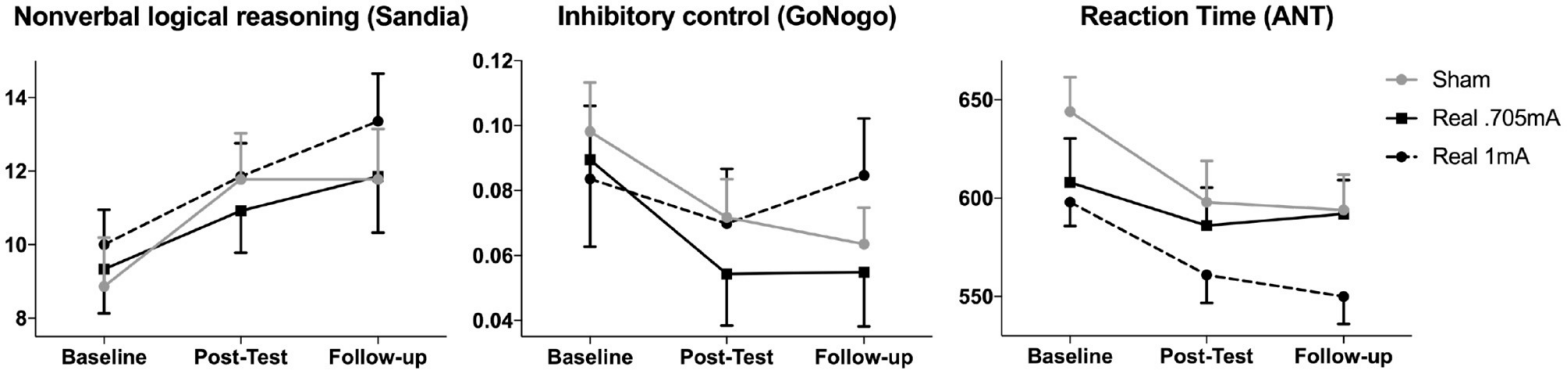
Cognitive performance over time. The main outcome measure non-verbal logical reasoning (Sandia) as well as inhibitory control (GoNogo false alarms) and reaction time (ANT) improved significantly over time across groups, but stimulation did not differentially affect this improvement. These improvements were driven by significant changes during the intervention (from baseline to post-test) in all three measures (mean and standard errors are shown).

Over time, participants significantly improved in the Sandia test of logical reasoning [*F*_(2,76)_ = 12.441, *p* < 0.001, $\eta_{\text{p}}^{2}$ = 0.247], yielded faster reaction times in the attention network test [*F*_(1.62,66.35)_ = 10.538, *p* < 0.001, $\eta_{\text{p}}^{2}$ = 0.204, adjusted by Greenhouse-Geisser] and in inhibitory control [*F*_(1.55,64.95)_ = 5.492, *p* = 0.011, $\eta_{\text{p}}^{2}$ = 0.116, adjusted by Greenhouse-Geisser] (Figure 2). *Post-hoc* tests using the Tukey HSD test indicated that this was driven by changes during the intervention from baseline to post-test in all three measures [Sandia: *p* = 0.002, 95% CI (−3.71, −0.92); reaction time: *p* = 0.002, 95% CI (12.72, 50.96); inhibitory control: *p* = 0.015, 95% CI (0.005, 0.045)] while no significant changes were observed after the end of the intervention from the post-test to the follow-up test [Sandia: *p* = 0.158, 95% CI (−2.25, 0.38); reaction time: *p* = 0.663, 95% CI (−14.77, 9.50); inhibitory control: *p* = 0.680, 95% CI (−0.009, 0.014)].

To complement the null findings on the interaction between time and group on the different outcome measures, we performed Bayesian analyses using the software, JASP (65) with default priors. First, we ran a mixed Bayesian ANOVA with Time as within-subjects factor and Group as between-subjects factor. We found that the null model was preferred to the interaction between time and group model, as the Bayes factors (BF_10_) were smaller than 0.173 thus providing at least a moderate support for the null hypothesis. However, in two cases the interaction term was preferred over the null model (Sandia non-verbal logical reasoning and ANT reaction time). Notably, in both cases this was due to the inclusion of the main effect of time that was the best predictor, showing an improvement in performance over time. In other words, the inclusion of the interaction between time and group to the main effect of time, benefited from the variance explained by the factor time, and reduced the strength of this model. Overall, these analyses provided at least moderate evidence in favor of the null hypothesis (Supplementary Results).

**Figure 3:**
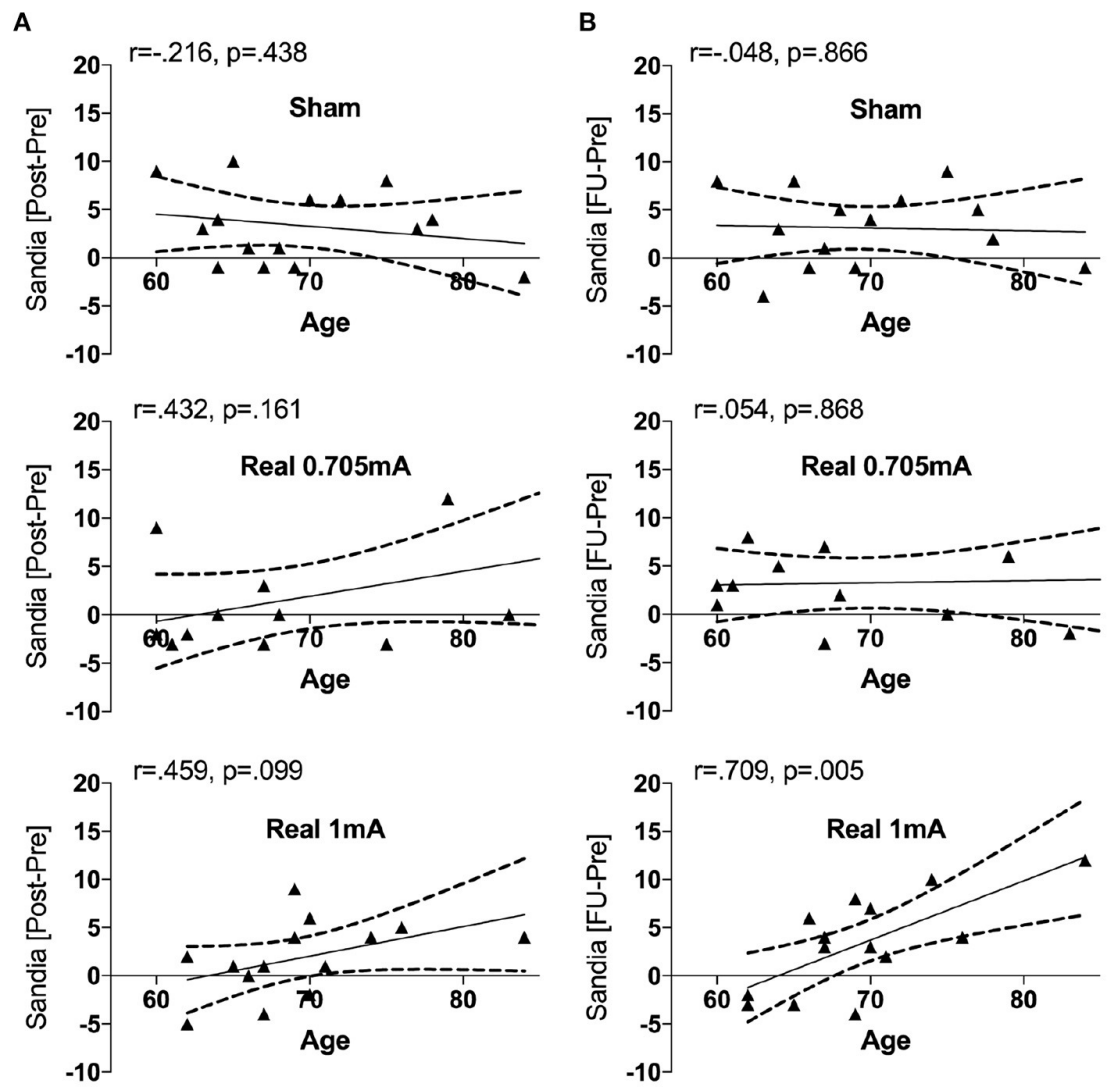
Correlations between the change in Sandia performance and age in the three intervention groups. Age was associated with improvement in performance in the group receiving 1 mA tRNS, indicating that older participants profited significantly more from tRNS than younger participants. During the intervention **(A)** this association was marginal, while it was significant from pre to follow-up **(B)**. The correlation in the group receiving 1 mA tRNS was significantly stronger than in the groups receiving 0.705 mA tRNS or sham stimulation.

In order to explore a possible influence of age, we entered this factor as a covariate. When entering *age* as a covariate, we found a significant interaction in the main outcome (Sandia) between time^*^stimulation [*F*_(4,70)_ = 3.11, *p* = 0.021, $\eta_{\text{p}}^{2}$ = 0.151] and between time^*^stimulation^*^age [*F*_(4,70)_ = 3.03, *p* = 0.023, $\eta_{\text{p}}^{2}$ = 0.148] fitting a linear function. At baseline, the Sandia was significantly correlated with age (*r* = −0.521, *p* < 0.001) indicating that older subjects showed lower performance in logical reasoning. The change from pre to post was marginally correlated with age in the group receiving 1 mA tRNS (*r* = 0.459, *p* = 0.099), while it was not significant in either the group receiving 0.705 mA tRNS (*r* = 0.432, *p* = 0.161) or sham (*r* = −0.216, *p* = 0.438) (Figure 3A). Similarly, the change from pre to follow-up was significantly correlated with age in the group receiving 1 mA tRNS (*r* = 0.709, *p* = 0.005), while it was not significant in either the group receiving 0.705 mA tRNS (*r* = 0.054, *p* = 0.868) or sham (*r* = −0.048, *p* = 0.866). The correlation in the 1 mA tRNS group was significantly stronger than in the sham group (*p* = 0.003) and marginally stronger than in the 0.705 tRNS group (*p* = 0.064) (Figure 3B).

We furthermore found a significant interaction in non-verbal working memory between time^*^stimulation [*F*_(4,80)_ = 3.19, *p* = 0.018, $\eta_{\text{p}}^{2}$ = 0.137] and time^*^stimulation^*^age [*F*_(4,80)_ = 3.16, *p* = 0.018, $\eta_{\text{p}}^{2}$ = 0.136]. This interaction fit a quadratic function indicating a decline from pre to post in all three groups, and improvement from post to follow-up only for the active tRNS conditions (see Table 2 and Supplementary Figure 2). The change from pre to post was significantly correlated with age in the group receiving sham tRNS (*r* = −0.635, *p* = 0.005), while it was not significant in either the group receiving 0.705 mA tRNS (*r* = −0.012, *p* = 0.968) or 1 mA tRNS (*r* = 0.181, *p* = 0.536). The correlation in the sham group was significantly different from the 1 mA tRNS group (*p* = 0.019) (Supplementary Figure 3A), indicating that in contrast to active tRNS, older participants in the sham group declined more than younger participants. The change from pre to follow-up was not significantly correlated with age in any of the groups (Supplementary Figure 3B).

### Engagement Questionnaire

The overall self-reported commitment with the computer-based CT as declared in the engagement questionnaire (Table 3) can be considered as medium. Subjects reported an overall high effort in playing the game and had to invest a lot of cognitive effort to follow instructions. They perceived the game as challenging but did not become so involved that they wanted to speak to the game or felt emotionally attached to it. They nevertheless tried their best while forgetting about everyday concerns during the training and not being distracted by things that were ongoing in their surroundings. However, they did not reach a point where they were able to play the game effortlessly, i.e., to a degree where they were unaware of even using controls. Finally, they were not disappointed when the training was over.

**Table 3:** Self-reported commitment with the CT.

**Questions game engagement**	**Mean**	**SD**
1. To what extent did the game hold your attention?	5.8	1.4
2. To what extent did you feel you were focused on the game?	5.9	1.3
**3. How much effort did you put into playing the game?**	**6.4**	**0.8**
**4. Did you feel that you were trying your best?**	**6.4**	**0.8**
5. To what extent did you lose track of time, e.g., did the game absorb your attention so that you were not bored?	5.4	1.6
6. To what extent did you feel consciously aware of being in the real world whilst playing?	4.6	1.9
**7. To what extent did you forget about your everyday concerns?**	**6.3**	**0.9**
8. To what extent were you aware of yourself in your surroundings?	5.0	1.8
9. To what extent did you notice events taking place around you?	4.2	1.8
**10. Did you feel the urge at any point to stop playing and see what was happening around you?**	**1.9**	**1.3**
11. To what extent did you feel that you were interacting with the game environment?	5.0	1.8
12. To what extent did you feel as though you were separated from your real-world environment?	4.1	1.9
13. To what extent did you feel that the game was something fun you were experiencing, rather than a task you were just doing?	3.8	2.1
14. To what extent was your sense of being in the game environment stronger than your sense of being in the real world?	3.6	2.1
**15. At any point did you find yourself become so involved that you were unaware you were even using controls, e.g., it was effortless?**	**2.4**	**1.7**
16. To what extent did you feel as though you were moving through the game according to your own will?	3.0	1.9
**17. To what extent did you find the game challenging?**	**6.3**	**0.8**
18. Were there any times during the game in which you just wanted to give up?	3.7	2.0
19. To what extent did you feel motivated while playing?	5.0	1.7
**20. To what extent did you find the game easy?**	**2.6**	**1.5**
21. To what extent did you feel like you were making progress toward the end of the game?	3.6	1.6
22. How well do you think you performed in the game?	3.3	1.4
**23. To what extent did you feel emotionally attached to the game?**	**2.0**	**1.4**
24. To what extent were you interested in seeing how the game's events would progress?	3.6	2.0
25. How much did you want to “win” the game?	5.6	1.8
26. Were you in suspense about whether or not you would do well in the game?	4.1	2.0
**27. At any point did you find yourself become so involved that you wanted to speak to the game directly?**	**2.3**	**1.7**
28. To what extent did you enjoy the graphics and the imagery?	3.4	2.0
29. How much would you say you enjoyed playing the game?	3.9	1.9
**30. When it ended, were you disappointed that the game was over?**	**2.3**	**1.7**
31. Would you like to play the game again?	3.3	2.1
32. How immersed did you feel?	3.7	1.9
33. Did you read all task instruction completely before starting each task? “No” (*n* = 9), “Yes” (*n* = 38)		
34. Did you understand the task instructions?	4.4	1.4
**35. How much mental effort did it take for you to follow the instructions?**	**6.3**	**0.9**

*Raw scores of all participants are presented. The visual analog scale ranged from 0 (“Not at all”) to 7 (“A lot”). Bold font indicates items with an average response above 6 or below 3*.

### Blinding and Side Effects

Apart from one participant who dropped out due to headache after the second intervention session (1 mA), the side effects questionnaire showed that all other participants tolerated the stimulation very well with only minor discomfort reported (i.e., itching and tingling comparable for all groups). The proportion of subjects who reported being stimulated actively did not differ by stimulation group $\chi_{\left(2,\text{N}=44\right)}^{2}$ = 0.799, *p* = 0.740. Three subjects (all from the group receiving 0.705 mA) were undecided whether they received real or sham stimulation. Overall, only 40% of participants identified correctly which type of stimulation they received [sham: 47% (9/19), active 1 mA: 43% (6/14), active 0.705 mA: 36% (4/14)]. This response is below chance level; we can therefore assume that blinding was effective. Interestingly, while 40% of all participants received sham stimulation, 51% of all participants believed they received sham stimulation, indicating a global negative expectation toward the protocol they received. This finding is also in accordance with the fact that tRNS is perceived less strongly than tDCS (66).

## Discussion

In the present study, conducted in a sample of healthy older adults, we found improvements in non-verbal logical reasoning (primary outcome measure to assess far transfer effects), inhibitory control and in reaction times (as measured with the ANT) in all intervention groups after 5 days of combined neurostimulation and CT targeting executive functions. These changes occurred during the intervention phase, while no further improvement was observed during follow-up. As all participants received CT, far transfer improvements in non-verbal logical reasoning could be due to both training effects (changes due to the treatment) as well as practice effects (changes due to repeated testing). Given that we used parallel tests to assess logical reasoning and did not find practice effects in any of the other cognitive tasks (even for tasks without parallel versions), training effects are likely to play the principal role. We moreover found improvements over time in inhibitory control, which indicates near transfer effects in this executive domain that was trained during the CT. No near transfer effects were found in working memory, an improvement, which we had expected to occur, given that we hypothesized that far transfer effects result from activity in common underlying brain networks. Neurostimulation (tRNS at 1 or 0.705 mA) did not significantly modulate other cognitive functions, although we found that age moderated the effects of tRNS on non-verbal working memory and logical reasoning over time. Importantly, the performance change in logical reasoning was significantly correlated with age in the group receiving 1 mA tRNS, indicating that older participants profited more from tRNS than younger participants. CT combined with tRNS, at least using the current of 1 mA, might be particularly effective in older adults. This effect could be explained via its main mechanism, stochastic resonance, which can mitigate the age-dependent increase in neuronal noise (50). However, other studies have suggested that tRNS might be less effective in older than younger adults (22). We used a scientifically rigorous double-blind, sham-controlled, randomized study design to investigate the questions put forth with as minimal experimental bias as possible and confirmed successful blinding of the subjects. However, several drawbacks likely contributed to the rejection of our main hypothesis. We discuss possible reasons leading to these findings to help improving the design of future tES studies and encourage researchers to consider specific requirements for the aging population. Table 4 contains a graphical representation of the possible factors that might be responsible for our findings.

**Table 4:** Possible explanations and solutions for future trials.

	**Possible explanation**	**Possible solutions/directions for future trials**
**Sample**	• Small sample size • High level of education and baseline performance • Large variability	• Investigate larger sample sizes • Select a sample more representative of the general aging population and/or with lower baseline performance (e.g., with subjective cognitive impairments) • Investigation of interindividual mechanisms and comparison of different protocols
**Study design**	• Low number of intervention sessions • Short follow-up (4 weeks)	• Longer intervention protocols • Longer follow-up (several months)
**tRNS**	• One-size-fits-all protocol (electrode positioning, stimulation intensity) • Low stimulation intensity • Belief of belonging to the sham group (both real and sham tRNS produce only little physical sensations)	• Personalized protocol of stimulation based on individual modeling, cognitive ability, and neurophysiology • Stimulation at higher intensities might be more efficient • Inform subjects that tRNS does not necessarily produce a sensation. Apply sham tDCS instead of sham tRNS at the beginning of both real and sham tRNS sessions in order to create a stronger sensation
**Cognitive training**	• Adaptive CT but not personalized • Low level of motivation / high frustration • Too challenging (high level of effort to play the game, difficulty understanding tasks) • Single subject training • Presentation of motivating but also negative feedback when performance was low	• Personalized CT (in accordance with baseline functionality) • CT should be designed to be appealing to older participants • CT should include a broad range of tasks and levels in order to ensure continuous motivation and challenge • Multiple-player options via networking technology (increase social aspect and achieve common goal, competition) • Negative feedback should be framed positively
**Cognitive assessment**	• High level of performance (possible ceiling effects) • Test instructions were partly challenging • No feedback on test performance or psychoeducation	• Test a broad range of functionality (avoid floor and ceiling effects) • Adapt test instructions to the needs of older participants (ample time to explain, include practice sessions to learn how to use technical devices) • Provide psychoeducation and feedback of performance to increase motivation

### General Reason

We might not have been able to detect the effect of active vs. sham brain stimulation in combination with CT due to the large inter-individual variation in the outcome of neuroenhancement interventions (67, 68), an effect which might be even larger in older adults due to the larger variability in age-related structural and functional brain changes.

### Reasons Related to the Sample

Our sample consisted of a very highly educated population with an average education duration of over 16 years that was enrolled from the Oxford area via the website *www.joindementiaresearch.co.uk*. We assume that this population likely operated at a high cognitive level, which could be associated with ceiling effects for both CT and stimulation effects, possibly limits the range for cognitive improvement after training (25–27) and is most likely not representative of the general older adult population of the UK. Moreover, a trend difference in baseline cognitive ability (as assessed with the MoCA) (Table 1), might have contributed to the non-significant effect of stimulation. Higher baseline performance is thought to be associated with less profit from cognitive interventions (25) and neurostimulation (26) and to induce less consistent stimulation gains (27).

### Reasons Related to the Gamification of the Training

A potential critical issue may have been the medium enjoyment of the training, as reported in the engagement questionnaire. The design and setting of the CT (a robot factory), originally developed for the use in young people, might not have been as appealing for older people, leading to task disengagement (69). Gamified cognitive tasks are supposed to be more motivating than traditional tasks, thus making the participant experience less effortful and potentially reducing drop-outs in longitudinal studies (70, 71). However, it was found that game mechanics might specifically reduce participant motivation in low-performing subjects, for example by presenting negative feedback, i.e., by visualizing low performance, especially when only a single player is present. This might lead to more de-motivation and/or frustration in elderly populations (71). Participants in the current study perceived the game as challenging and reported an overall high effort to succeed with the tasks and follow the instructions, which possibly prevented them from becoming entirely involved and immersed in the game.

### Reasons Related to Cognitive Testing

The high level of education and baseline performance might have led to ceiling effects in some of the tasks. However, some of the cognitive tests, particularly computerized tasks that require prolonged practice time, might have partly been too challenging even for this population. For older adults that are not used to using a computer and a mouse this possibly represented a challenge *per se*, despite providing ample practice time before each task.

### Reasons Related to Stimulation

The null effects might also be due to the stimulation protocol and setup used. For older adults an intensity of ≤ 1 mA might not be sufficient to modulate DLPFC activity given that the stimulated brain areas contain more cerebrospinal liquid resulting in a reduced current intensity in the brain and a possible change in the conduction of current vectors (72). Another possible explanation for the negative finding in our study could be the size of the electrodes (3.14 cm^2^, round electrodes); the behavioral effects of tES appear to be also critically dependent on the position of the return electrode. Our study used smaller active electrodes with a circular surface compared to many previous studies that stimulated the same area of interest using tES with larger or non-circular stimulation electrodes. Moreover, five stimulation sessions might have been insufficient to open a window of plasticity and trigger neurophysiological changes resulting in cognitive enhancement. Indeed, a recent study investigating combined tDCS and decision-making training over 5 days in older adults also failed to find a significant effect (73). In addition, our stimulation sessions were spread across 2 weeks (blocks of 3 and 2 days) and did not take place on 5 consecutive days. This may have affected tRNS efficacy.

### Neurobiological Reasons

The neurobiological mechanisms underlying tES effects are complex and involve changes at multiple levels, ranging from the cellular level to the modulation of intrinsic network dynamics across the brain. Functional reorganization due to aging, particularly in frontal areas of the brain, may partly explain why tES effects are generally variable across studies. Frontal regions are core hubs in working memory processing (74) and are known to be subjected to age-related changes in activation patterns as described in the PASA (posterior-anterior shift in aging) model (75). This model postulates that prefrontal over-activity might be a compensatory effort to mask age-related decline. Dependent on the stage of age-related changes, tES applied over frontal areas could therefore be used to support the primary activation pattern or compensatory processes. However, the rather large variability in response to brain stimulation is also increasingly investigated and discussed in young healthy populations (76, 77). Recent studies demonstrated that by using a combination of neurophysiological and neuroimaging methods with neurostimulation we can shed light on interindividual mechanisms that drive response variability (16, 22, 24, 46, 78, 79) and use this knowledge to improve stimulation efficacy (80). Associated with this, we also need to reconsider the practice of investigating questions in another than the target population. The simplistic view that similar protocols for brain-based interventions, which are effective in treating impaired patients or modulate functions in young healthy student populations, can be used to enhance the cognitive performance of healthy older subjects or to prevent future cognitive disorders, cannot be held up.

Finally, we would like to draw attention to ethical concerns when applying stimulation to improve functions in healthy subjects. The current study covered a broad range of cognitive functions besides the executive functions that were targeted with the training. As previously suggested (81), and demonstrated (82, 83), enhancement through NIBS might be a zero-sum proposition, where the enhancement of a cognitive or motor function comes at a cost of other abilities. The possibility that enhancing some cognitive or motor abilities in healthy older individuals could be at the expense of other abilities has clear ethical implications and is an important issue in need of systematic evaluation (84). We attempted to address this issue in the cognitive domain, but did not assess cross-domain functions (e.g., motor functions).

Notably, in this research area we find a strong association between significant results and publication: studies applying brain stimulation reporting positive or significant results are more likely to be published, and have mostly not been pre-registered. The pre-registration of the experimental design and analysis aims to counteract publication bias by anticipating the review process before the data is collected, in order to have reviewers focus on the importance of the research questions without looking at the findings ahead of time (85). Moreover, to date, funding is often not sufficient to investigate full-factorial designs and longer follow up visits.

To conclude, study protocols should acknowledge the differences between target populations, need to be specifically designed to fit the motivational preferences and baseline abilities, and take into account neurophysiological and anatomical features of the target population.

We believe that research on combined tES and CT remains a fundamentally important goal to counteract age-related cognitive decline with non-invasive means. Studies should compare more established stimulation protocols, such as tDCS, with novel protocols in order to clarify factors that determine tES responsiveness in aging and apply longer trainings. We would like to encourage future studies to assess larger sample sizes with training tasks designed in accordance with specific preferences for older people as well as underlying neurophysiological parameters to systematically expand our knowledge in this area to understand the neural mechanism that induce cognitive preservation and enhancement in the elderly.

## Data Availability Statement

The raw data supporting the conclusions of this article will be made available by the authors, without undue reservation.

## Ethics Statement

The studies involving human participants were reviewed and approved by Ethics committee of the University of Oxford. The patients/participants provided their written informed consent to participate in this study.

## Author Contributions

A-KB and RCK designed the study. MB, LD, JW, AB, and A-KB collected the data. MB, RCK, NZ, and A-KB analyzed the data. MB, LD, and JW prepared the initial draft report. A-KB edited the report, which was critically reviewed by all authors.

## Conflict of Interest

RC serves on the scientific advisory boards for Neuroelectrics and Innosphere and filed a UK Patent Application Number 2000874.4 (“method for obtaining personalized parameters for transcranial stimulation, transcranial system, method of applying transcranial stimulation”). This patent does not relate to this work. The remaining authors declare that the research was conducted in the absence of any commercial or financial relationships that could be construed as a potential conflict of interest.
