# Supplementary material for: The Effect of Transcranial Random Noise Stimulation on Cognitive Training Outcome in Healthy Aging
Source: Front Neurol. 2021 Mar 9;12:625359. doi: 10.3389/fneur.2021.625359 (PMC7985554; doi:10.3389/fneur.2021.625359)
Supplement: Supplementary file 1 [file Data_Sheet_1.docx]

**Supplementary material**

**Supplementary figure 1**

**4-item working memory task.** Participants had to rotate a probe bar to match the orientation of the same-coloured bar from the stimulus sequence (any colour).

**
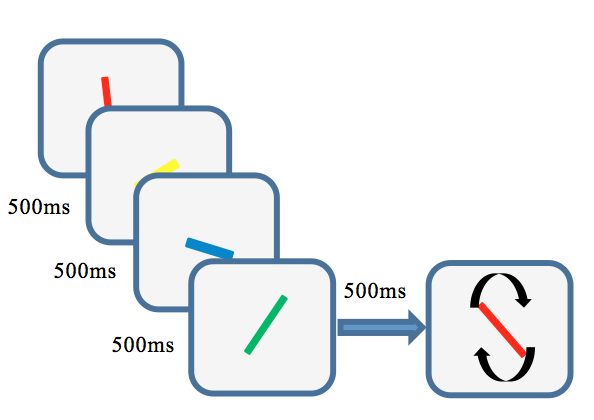
**

**Supplementary figure 2**

**Cognitive performance in nonverbal working memory.** A significant interaction in nonverbal working memory between *time* and *stimulation* when entering *age* as a covariate indicated a decline from pre to post in all intervention groups, and improvement from post to follow-up only for the active tRNS conditions (mean and standard errors are shown).

**
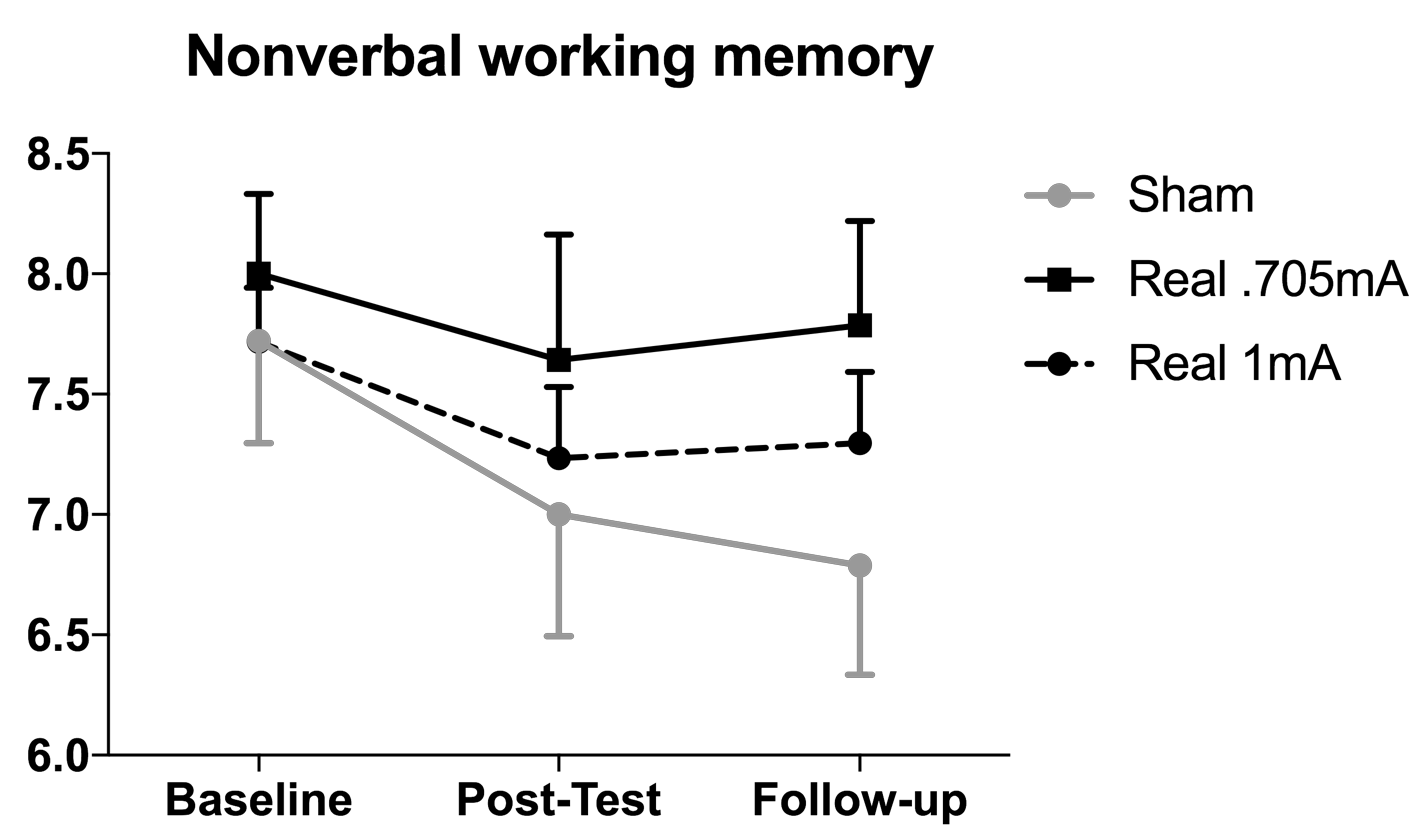
**

**Supplementary figure 3:**

**Correlations between the change in nonverbal working memory performance and age in the three intervention groups.** Age was significantly associated with a decline in performance from pre to post (panel A) in the group receiving sham tRNS, indicating that older participants declined significantly more during sham stimulation than younger participants. The correlation in the group receiving sham tRNS was significantly stronger than in the group receiving 1mA tRNS. After the intervention (panel B) the association between age and performance change was not significant.

**
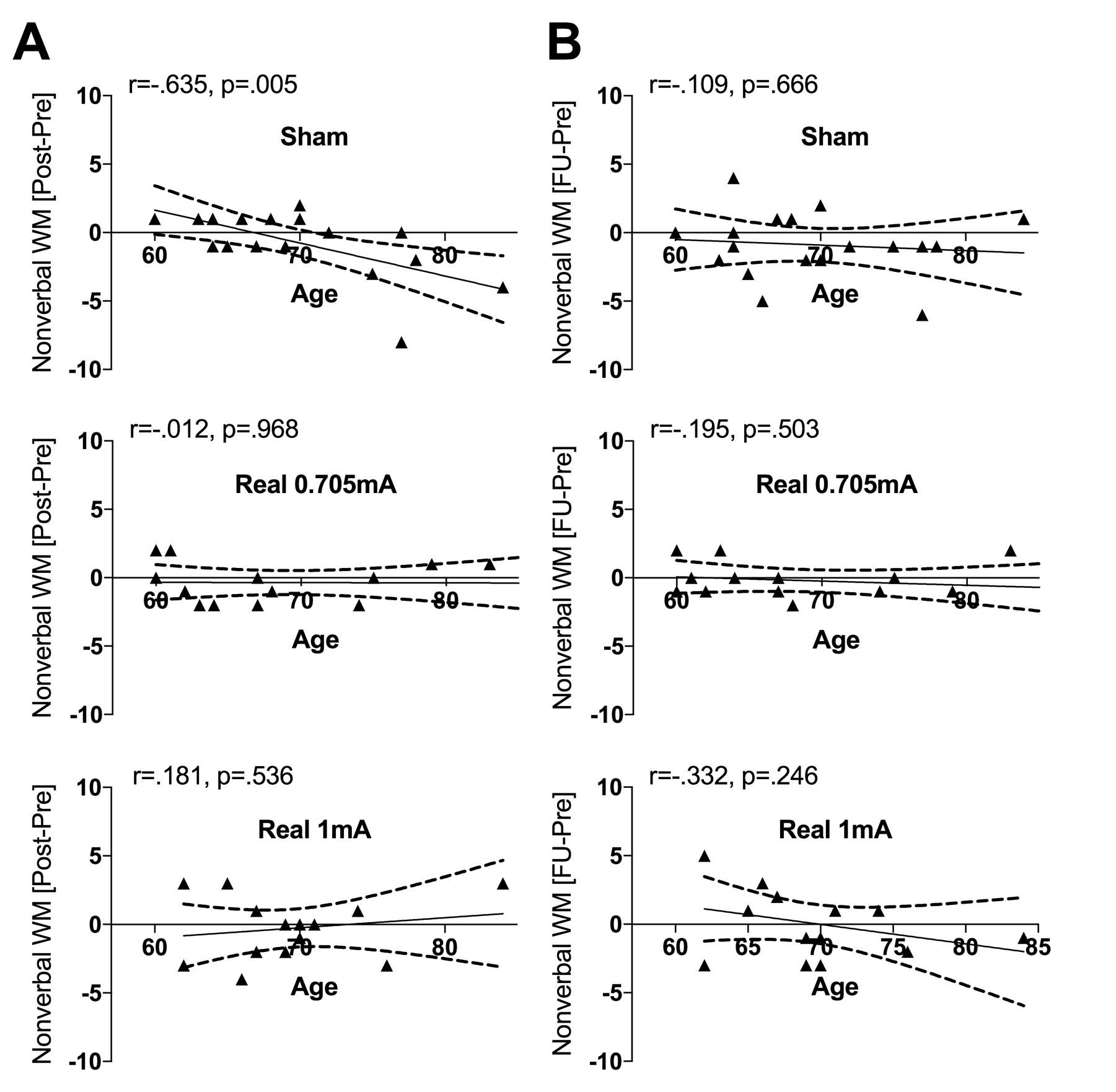
**

**Supplementary results (Bayesian analyses)**

## **Phonemic fluency (2 min)**

| Model Comparison | | | | | | | | | | | |
| --- | --- | --- | --- | --- | --- | --- | --- | --- | --- | --- | --- |
| Models | | P(M) | | P(M\|data) | | BF _M_ | | BF _10_ | | error % | |
| Null model (incl. subject) |  | 0.200 |  | 0.567 |  | 5.241 |  | 1.000 |  |  |  |
| STIM |  | 0.200 |  | 0.214 |  | 1.088 |  | 0.377 |  | 1.044 |  |
| RM Factor 1 |  | 0.200 |  | 0.156 |  | 0.741 |  | 0.276 |  | 1.651 |  |
| RM Factor 1 + STIM |  | 0.200 |  | 0.057 |  | 0.244 |  | 0.101 |  | 1.344 |  |
| RM Factor 1 + STIM + RM Factor 1  ✻  STIM |  | 0.200 |  | 0.005 |  | 0.021 |  | 0.009 |  | 1.631 |  |
|  | | | | | | | | | | | |
| Note.  All models include subject. Stim: Stimulation; RM: repeated measures of the factor time. | | | | | | | | | | | |

## **Semantic fluency (2 min)**

| Model Comparison | | | | | | | | | | | |
| --- | --- | --- | --- | --- | --- | --- | --- | --- | --- | --- | --- |
| Models | | P(M) | | P(M\|data) | | BF _M_ | | BF _10_ | | error % | |
| Null model (incl. subject) |  | 0.200 |  | 0.638 |  | 7.061 |  | 1.000 |  |  |  |
| STIM |  | 0.200 |  | 0.200 |  | 0.999 |  | 0.313 |  | 1.040 |  |
| RM Factor 1 |  | 0.200 |  | 0.115 |  | 0.520 |  | 0.180 |  | 0.789 |  |
| RM Factor 1 + STIM |  | 0.200 |  | 0.037 |  | 0.152 |  | 0.057 |  | 1.911 |  |
| RM Factor 1 + STIM + RM Factor 1  ✻  STIM |  | 0.200 |  | 0.010 |  | 0.041 |  | 0.016 |  | 1.487 |  |
|  | | | | | | | | | | | |
| Note.  All models include subject. Stim: Stimulation; RM: repeated measures of the factor time. | | | | | | | | | | | |

## **Verbal short-term memory**

| Model Comparison | | | | | | | | | | | |
| --- | --- | --- | --- | --- | --- | --- | --- | --- | --- | --- | --- |
| Models | | P(M) | | P(M\|data) | | BF _M_ | | BF _10_ | | error % | |
| Null model (incl. subject) |  | 0.200 |  | 0.332 |  | 1.986 |  | 1.000 |  |  |  |
| STIM |  | 0.200 |  | 0.350 |  | 2.153 |  | 1.055 |  | 1.777 |  |
| RM Factor 1 + STIM |  | 0.200 |  | 0.162 |  | 0.772 |  | 0.488 |  | 4.739 |  |
| RM Factor 1 |  | 0.200 |  | 0.146 |  | 0.684 |  | 0.440 |  | 0.664 |  |
| RM Factor 1 + STIM + RM Factor 1  ✻  STIM |  | 0.200 |  | 0.010 |  | 0.042 |  | 0.032 |  | 2.246 |  |
|  | | | | | | | | | | | |
| Note.  All models include subject. Stim: Stimulation; RM: repeated measures of the factor time. | | | | | | | | | | | |

## **Verbal working memory**

| Model Comparison | | | | | | | | | | | |
| --- | --- | --- | --- | --- | --- | --- | --- | --- | --- | --- | --- |
| Models | | P(M) | | P(M\|data) | | BF _M_ | | BF _10_ | | error % | |
| Null model (incl. subject) |  | 0.200 |  | 0.563 |  | 5.146 |  | 1.000 |  |  |  |
| STIM |  | 0.200 |  | 0.242 |  | 1.278 |  | 0.430 |  | 0.420 |  |
| RM Factor 1 |  | 0.200 |  | 0.131 |  | 0.603 |  | 0.233 |  | 0.681 |  |
| RM Factor 1 + STIM |  | 0.200 |  | 0.058 |  | 0.246 |  | 0.103 |  | 1.027 |  |
| RM Factor 1 + STIM + RM Factor 1  ✻  STIM |  | 0.200 |  | 0.006 |  | 0.025 |  | 0.011 |  | 1.608 |  |
|  | | | | | | | | | | | |
| Note.  All models include subject. Stim: Stimulation; RM: repeated measures of the factor time. | | | | | | | | | | | |

## **Nonverbal short-term memory**

| Model Comparison | | | | | | | | | | | |
| --- | --- | --- | --- | --- | --- | --- | --- | --- | --- | --- | --- |
| Models | | P(M) | | P(M\|data) | | BF _M_ | | BF _10_ | | error % | |
| Null model (incl. subject) |  | 0.200 |  | 0.684 |  | 8.640 |  | 1.000 |  |  |  |
| STIM |  | 0.200 |  | 0.174 |  | 0.840 |  | 0.254 |  | 0.533 |  |
| RM Factor 1 |  | 0.200 |  | 0.110 |  | 0.492 |  | 0.160 |  | 0.708 |  |
| RM Factor 1 + STIM |  | 0.200 |  | 0.028 |  | 0.115 |  | 0.041 |  | 0.819 |  |
| RM Factor 1 + STIM + RM Factor 1  ✻  STIM |  | 0.200 |  | 0.005 |  | 0.022 |  | 0.008 |  | 1.325 |  |
|  | | | | | | | | | | | |
| Note.  All models include subject. Stim: Stimulation; RM: repeated measures of the factor time. | | | | | | | | | | | |

## **Nonverbal working memory**

| Model Comparison | | | | | | | | | | | |
| --- | --- | --- | --- | --- | --- | --- | --- | --- | --- | --- | --- |
| Models | | P(M) | | P(M\|data) | | BF _M_ | | BF _10_ | | error % | |
| Null model (incl. subject) |  | 0.200 |  | 0.676 |  | 8.349 |  | 1.000 |  |  |  |
| STIM |  | 0.200 |  | 0.166 |  | 0.796 |  | 0.245 |  | 0.569 |  |
| RM Factor 1 |  | 0.200 |  | 0.124 |  | 0.566 |  | 0.183 |  | 0.575 |  |
| RM Factor 1 + STIM |  | 0.200 |  | 0.031 |  | 0.129 |  | 0.046 |  | 1.683 |  |
| RM Factor 1 + STIM + RM Factor 1  ✻  STIM |  | 0.200 |  | 0.003 |  | 0.012 |  | 0.004 |  | 1.785 |  |
|  | | | | | | | | | | | |
| Note.  All models include subject. Stim: Stimulation; RM: repeated measures of the factor time. | | | | | | | | | | | |

## **Nonverbal logical reasoning (Sandia)**

| Model Comparison | | | | | | | | | | | |
| --- | --- | --- | --- | --- | --- | --- | --- | --- | --- | --- | --- |
| Models | | P(M) | | P(M\|data) | | BF _M_ | | BF _10_ | | error % | |
| Null model (incl. subject) |  | 0.200 |  | 5.248e -4 |  | 0.002 |  | 1.000 |  |  |  |
| RM Factor 1 |  | 0.200 |  | 0.766 |  | 13.083 |  | 1459.435 |  | 1.716 |  |
| RM Factor 1 + STIM |  | 0.200 |  | 0.211 |  | 1.071 |  | 402.438 |  | 2.791 |  |
| RM Factor 1 + STIM + RM Factor 1  ✻  STIM |  | 0.200 |  | 0.022 |  | 0.091 |  | 42.528 |  | 5.477 |  |
| STIM |  | 0.200 |  | 1.300e -4 |  | 5.201e -4 |  | 0.248 |  | 2.230 |  |
|  | | | | | | | | | | | |
| Note.  All models include subject. Stim: Stimulation; RM: repeated measures of the factor time. | | | | | | | | | | | |

## **ANT reaction time**

| Model Comparison | | | | | | | | | | | |
| --- | --- | --- | --- | --- | --- | --- | --- | --- | --- | --- | --- |
| Models | | P(M) | | P(M\|data) | | BF _M_ | | BF _10_ | | error % | |
| Null model (incl. subject) |  | 0.200 |  | 5.306e -4 |  | 0.002 |  | 1.000 |  |  |  |
| RM Factor 1 |  | 0.200 |  | 0.446 |  | 3.226 |  | 841.382 |  | 0.656 |  |
| RM Factor 1 + STIM |  | 0.200 |  | 0.353 |  | 2.182 |  | 665.178 |  | 1.821 |  |
| RM Factor 1 + STIM + RM Factor 1  ✻  STIM |  | 0.200 |  | 0.200 |  | 0.998 |  | 376.378 |  | 1.743 |  |
| STIM |  | 0.200 |  | 3.875e -4 |  | 0.002 |  | 0.730 |  | 0.760 |  |
|  | | | | | | | | | | | |
| Note.  All models include subject. Stim: Stimulation; RM: repeated measures of the factor time. | | | | | | | | | | | |

## **Inhibitory control (false alarms)**

| Model Comparison | | | | | | | | | | | |
| --- | --- | --- | --- | --- | --- | --- | --- | --- | --- | --- | --- |
| Models | | P(M) | | P(M\|data) | | BF _M_ | | BF _10_ | | error % | |
| Null model (incl. subject) |  | 0.200 |  | 0.085 |  | 0.374 |  | 1.000 |  |  |  |
| RM Factor 1 |  | 0.200 |  | 0.650 |  | 7.443 |  | 7.614 |  | 0.903 |  |
| RM Factor 1 + STIM |  | 0.200 |  | 0.196 |  | 0.977 |  | 2.297 |  | 3.111 |  |
| RM Factor 1 + STIM + RM Factor 1  ✻  STIM |  | 0.200 |  | 0.043 |  | 0.181 |  | 0.506 |  | 4.906 |  |
| STIM |  | 0.200 |  | 0.025 |  | 0.101 |  | 0.288 |  | 2.372 |  |
|  | | | | | | | | | | | |
| Note.  All models include subject. Stim: Stimulation; RM: repeated measures of the factor time. | | | | | | | | | | | |

## **4-item WM task**

| Model Comparison | | | | | | | | | | | |
| --- | --- | --- | --- | --- | --- | --- | --- | --- | --- | --- | --- |
| Models | | P(M) | | P(M\|data) | | BF _M_ | | BF _10_ | | error % | |
| Null model (incl. subject) |  | 0.200 |  | 0.329 |  | 1.961 |  | 1.000 |  |  |  |
| STIM |  | 0.200 |  | 0.312 |  | 1.815 |  | 0.949 |  | 1.300 |  |
| RM Factor 1 |  | 0.200 |  | 0.154 |  | 0.727 |  | 0.468 |  | 0.802 |  |
| RM Factor 1 + STIM |  | 0.200 |  | 0.148 |  | 0.696 |  | 0.450 |  | 1.061 |  |
| RM Factor 1 + STIM + RM Factor 1  ✻  STIM |  | 0.200 |  | 0.057 |  | 0.241 |  | 0.173 |  | 1.113 |  |
|  | | | | | | | | | | | |
| Note.  All models include subject. Stim: Stimulation; RM: repeated measures of the factor time. | | | | | | | | | | | |
